# Development and Evaluation of ‘Briefing Notes’ as a Novel Knowledge Translation Tool to Aid the Implementation of Sex/Gender Analysis in Systematic Reviews: A Pilot Study

**DOI:** 10.1371/journal.pone.0110786

**Published:** 2014-11-05

**Authors:** Marion Doull, Vivian Welch, Lorri Puil, Vivien Runnels, Stephanie E. Coen, Beverley Shea, Jennifer O’Neill, Cornelia Borkhoff, Sari Tudiver, Madeline Boscoe

**Affiliations:** 1 School of Population and Public Health, Faculty of Medicine, University of British Columbia, Vancouver, British Columbia, Canada; 2 Bruyere Research Institute, University of Ottawa, Ottawa, Ontario, Canada; 3 Therapeutics Initiative, University of British Columbia, Vancouver, British Columbia, Canada; 4 Globalization and Health Research Unit, Institute of Population Health, University of Ottawa, Ottawa, Ontario, Canada; 5 Department of Geography, Queen’s University, Mackintosh-Corry Hall, Kingston, Ontario, Canada; 6 Centre for Global Health, Institute of Population Health, University of Ottawa, Ottawa, Ontario, Canada; 7 Division of Pediatric Medicine, Child Health Evaluative Sciences, The Hospital for Sick Children, Toronto, Ontario, Canada; 8 Researcher/Consultant on Gender and Health, Ottawa, Ontario, Canada; 9 Reach Community Health Centre, Vancouver, British Columbia, Canada; University Hospital Lausanne, Switzerland

## Abstract

**Background:**

There is increasing recognition of sex/gender differences in health and the importance of identifying differential effects of interventions for men and women. Yet, to whom the research evidence *does* or *does not* apply, with regard to sex/gender, is often insufficiently answered. This is also true for systematic reviews which synthesize results of primary studies. A lack of analysis and reporting of evidence on sex/gender raises concerns about the applicability of systematic reviews. To bridge this gap, this pilot study aimed to translate knowledge about sex/gender analysis (SGA) into a user-friendly ‘briefing note’ format and evaluate its potential in aiding the implementation of SGA in systematic reviews.

**Methods:**

Our Sex/Gender Methods Group used an interactive process to translate knowledge about sex/gender into briefing notes, a concise communication tool used by policy and decision makers. The briefing notes were developed in collaboration with three Cochrane Collaboration review groups (HIV/AIDS, Hypertension, and Musculoskeletal) who were also the target knowledge users of the briefing notes. Briefing note development was informed by existing systematic review checklists, literature on sex/gender, in-person and virtual meetings, and consultation with topic experts. Finally, we held a workshop for potential users to evaluate the notes.

**Results:**

Each briefing note provides tailored guidance on considering sex/gender to reviewers who are planning or conducting systematic reviews and includes the rationale for considering sex/gender, with examples specific to each review group’s focus. Review authors found that the briefing notes provided welcome guidance on implementing SGA that was clear and concise, but also identified conceptual and implementation challenges.

**Conclusions:**

Sex/gender briefing notes are a promising knowledge translation tool. By encouraging sex/gender analysis and equity considerations in systematic reviews, the briefing notes can assist systematic reviewers in ensuring the applicability of research evidence, with the goal of improved health outcomes for diverse populations.

## Introduction

Sex/gender analysis is a framework used to guide researchers in assessing whether interventions have meaningful differential effects for men and women or, girls, and boys. Sex refers to the biological, genetic and physiological processes that generally distinguish males and females. Gender refers to the roles, relationships, relative power and other traits that societies generally ascribe to women, men and people of diverse gender identities (e.g., transgender persons). The term sex/gender is used here to highlight the ways in which the concepts of sex and gender are entangled, multidimensional and interactive [1], [2], [3]. In the context of health, sex/gender analysis can help to illuminate differences in baseline risk of health outcomes, relative effects of a condition or intervention, economic considerations, and acceptability or preferences related to implementation of an intervention [3], [4], [5], [6]. Indeed, the application of sex/gender analysis to health research has revealed that men and women often exhibit different vulnerabilities, symptoms and responses to treatments [7], [8], [9], [10], [11], [12]. There is now widespread international recognition of the importance of implementing sex/gender analysis in health research. To date stakeholders include research funders (e.g., the Canadian Institutes of Health Research), biomedical journal editors (e.g., the European Association of Scientific Editors; the Journal of the International AIDS Society; the Journal of the National Cancer Institute), and organizations such as the Institute of Medicine in the U.S and the Public Health Agency of Canada [13], [14], [15], [16], [17], [18].

Systematic reviews “are summaries of research evidence that address a clearly formulated question using systematic and explicit methods to identify, select, and critically appraise relevant research, and to collect and analyse data from the studies that are included in the review. Statistical methods (meta-analysis) may or may not be used to analyse and summarise the results of the included studies (p.S1)” [19]. Systematic reviews are increasingly accepted as important evidence-based decision-making aids [19]. However, decision-makers cite the lack of equity considerations (including sex/gender) as barriers to using systematic reviews [20], [21]. The lack of analysis and lack of reporting of evidence about sex/gender in systematic reviews raise scientific and ethical concerns [22], [23], [24].

Barriers to implementing sex/gender analysis in systematic reviews are manifold [25]. Evidence suggests that there is a general lack of understanding of the concepts of sex and gender, how they are interrelated, whether and how they affect health interventions [3], [26], [27], [28]. Other barriers include: limited access to sex-disaggregated data, issues with data quality and reporting, challenges related to measuring and analysing gender and, a lack of guidance on methods [25], [29], [30], [31].

More recently, however, because many funding agencies now have policies mandating that both men and women be included in clinical trials and that results for men and women be reported and interpreted separately, more researchers have begun to conduct sex/gender analysis [32], [33]. Systematic reviewers, most notably those working within the Cochrane Collaboration, are also increasingly examining the related question “To whom does this evidence apply?” in their assessment of the quality of available evidence. However, this is an emerging area of inquiry and there continues to be a lack of exemplar reviews that address sex/gender considerations. When reviewers have addressed sex/gender their conclusions have often been contingent on methodological or data limitations. For example, a systematic review of tobacco and smoking cessation interventions found some differences in intervention effects for girls and boys [34]. The authors reported that school-based restrictions may be more effective for girls whereas increases in the price of tobacco products may have more influence on boys; however, these findings were often from single or methodologically weak studies [34]. Further, in a systematic review on quality of life after total hip or total knee arthroplasty, men appeared to benefit more from the intervention but the authors stated that their conclusion was tempered by the few studies that addressed this issue [35]. These reviews underscore the importance of considering both similarities and differences across sex/gender as well as determining the quality of the available evidence.

Since 2005, our research group [Sex/Gender Methods Group: http://equity.cochrane.org/sex-and-gender-analysis] has been working to increase the awareness of, and foster uptake of, sex/gender analysis in systematic reviews. In 2012, we joined forces with the Campbell and Cochrane Equity Methods Group [36], [37] to specifically advance an understanding of sex/gender related processes, including their relation to health equity. As a result of numerous planning and knowledge gathering activities including sex/gender analysis workshops at several Cochrane Collaboration Conferences and an in-depth two day methodology meeting with sex/gender and systematic review experts, we identified a need to translate evidence about sex/gender to systematic reviewers. We decided to adopt briefing notes to complete this task because they allow for the synthesis of evidence and seemed well suited to the targeted users of systematic reviews. Briefing notes, communication tools commonly used by policy and decision makers, concisely describe an issue along with pertinent evidence, options and recommended actions in a user friendly format intended to increase awareness and uptake [38], [39]. They are sometimes referred to as ‘evidence briefs’ and are widely used by leading health research groups such as National Institute for Health and Care Excellence (NICE, UK) to concisely communicate evidence for decision making [40]. This article describes the development, pilot testing and evaluation of sex/gender briefing notes to translate evidence about how to consider sex/gender in systematic reviews.

## Materials and Methods

We adopted the definition of knowledge translation (KT) put forth by the Canadian Institutes of Health Research as “a dynamic and iterative process that includes synthesis, dissemination, exchange and ethically-sound application of knowledge” [41]. Our overall approach to KT was informed by ‘diffusion of innovations theory’ as elaborated by Rogers and Estabrooks [42], [43]. Diffusion of innovations theory has four main elements comprising “the process by which (1) an innovation (2) is communicated through certain channels (3) over time, (4) among the members of a social system (p.11)” [42]. The innovation in this context is a novel approach to systematic reviews in which sex/gender are to be systematically considered and included in the identification, appraisal, synthesis and presentation of the evidence. The planned means of communication are briefing notes that highlight and synthesize some of the evidence surrounding the importance of sex/gender analysis, and provide guidance on implementing this analysis into systematic reviews. The targeted users of the innovation are the editors and authors of the HIV/AIDS, Hypertension, and Musculoskeletal Cochrane Review Groups (i.e., social system members). Notably, we tried to ensure that the content of the briefing notes was coherent with users’ values, namely, that the best evidence should be synthesized in a manner that improves the quality of the knowledge base, including enhancement of its applicability [44], [45]. Our research team’s knowledge of systematic reviews, expertise in sex/gender analysis and existing relationships with review groups, positioned the group well to design the briefing notes to meet knowledge user needs. We anticipate that these factors will help increase the likelihood of the effective use of sex/gender analysis in systematic reviews over time [43], [46], [47].

The briefing notes developed for this project are designed to prompt reviewers to think through points where sex/gender should be considered along the trajectory of a systematic review (e.g., in background/rationale; methods; and conclusions). The briefing notes contain four main sections: 1) introduction to the issue; 2) defining sex and gender (concepts and description of sex/gender analysis); 3) why addressing sex and gender in systematic reviews matters (outline of evidence on sex/gender differences and similarities related to the specific field); and 4) what can and should be done (provision of methodological guidance) [see: Appendix S1 for a sample briefing note].

We worked with the Cochrane Collaboration HIV/AIDS, Hypertension, and Musculoskeletal Review Groups. These three systematic review groups were chosen for the pilot study because substantive evidence in each of these areas demonstrates differences in benefit and/or harm of interventions on the basis of sex/gender. Over a period of four months (Jan. 2012–Apr. 2012), our team of authors who have expertise in methodology, sex/gender analysis, systematic reviews, policy and knowledge translation, and additional clinical experts from the three review groups, worked together on the draft briefing notes.

We developed the first three sections of the briefing notes by initially reviewing existing information and guidance on sex/gender analysis as well as literature specific to the fields of HIV/AIDS, hypertension and musculoskeletal disorders [3], [4], [5], [48], [49]. A topic-specific literature scan was conducted for each of the three briefing notes (i.e., HIV/AIDS, Hypertension, Musculoskeletal) to identify areas where sex/gender are or might be relevant to health outcomes. For example, the HIV/AIDS briefing note highlights women and men’s differential risks for exposure, risks of seroconversion once exposed and responses to therapy including adverse effects. The musculoskeletal note highlights the difference in prevalence for some musculoskeletal disorders for men and women often reflecting underlying pathophysiologic mechanisms. The hypertension note provides examples related to the differing manifestation, outcomes and prevalence of hypertension among women and men. To develop the fourth section on methods (“what can and should be done”), we built on existing structured guidance for systematic reviewers by appraising four current checklists for systematic reviews: 1) the Preferred Reporting Items for Systematic Reviews and Meta-analyses (PRISMA)-Equity reporting guidelines [50]; 2) AMSTAR: a measurement tool to assess the methodological quality of systematic reviews. [51]; 3) Methodological Standards for the Conduct of Cochrane Intervention Reviews [52]; and (4) the Campbell and Cochrane Equity Methods Group Equity Checklist [53].

The Cochrane Review Groups suggested that topic-specific examples were needed for each briefing note. We identified Cochrane and non-Cochrane peer-reviewed published systematic reviews in each topic area where elements of the reviews addressed an aspect of sex/gender. Reviews were chosen in several ways: by seeking review nominations from our collaborators in each of the review groups; searching for sex and gender in the Cochrane Database of Systematic Reviews; using the Montori search filter for sensitivity in searching for systematic reviews [54] and using the text words “sex” and “gender” in PubMed. Significantly, we did not find exemplar reviews that served as a model for integrating sex/gender analysis but identified examples of reviews where some limited consideration was given to sex/gender issues.

After a period of feedback and revision from the three collaborating review groups we moved to evaluate the briefing notes with a broader group of users. We conducted a workshop at the 2012 Canadian Cochrane Symposium and invited user feedback on the briefing notes. The Canadian Cochrane Symposium provided a very useful setting to evaluate the briefing notes as it gathers a diverse array of participants involved in systematic reviews including systematic review authors, methodologists, researchers and users of systematic reviews such as policy makers and health care practitioners. The workshop was open to all conference attendees with all levels of experience in systematic reviews in order to ensure that the comprehensibility of the briefing notes were evaluated by experts and non-experts. Participants self-selected to attend.

The workshop evaluation was comprised of a total of 17 questions focused on the content, readability and comprehensiveness of the briefing notes. Questions specifically addressed whether participants found each section clear and easy to understand (e.g., “the concepts of sex/gender are clearly defined”); readable (e.g., “the format of the briefing note makes it easy to read”); whether the examples provided were helpful to illustrate the application of sex/gender analysis to systematic reviews; and whether the briefing note overall was considered credible (e.g., “the briefing note is credible”). Participants were asked to evaluate questions using a 7-point Likert scale and were invited to enter comments to support their scores throughout. Median scores and ranges were calculated for each question with higher scores indicating more positive endorsement of the briefing note (highest score 7). Additional closed ended questions asked about participant expertise and knowledge of sex/gender and systematic reviews. A simple content analysis of responses was conducted and similar responses were grouped according to each evaluation question. Examples of responses are quoted verbatim below to illustrate respondents’ insights into the key areas evaluated.

## Results

In total, 19 workshop participants with backgrounds in research, clinical practice, community health, education and health policy evaluated the briefing notes. Participants had a range of experience with systematic reviews (no experience to 10 years) and the majority were “somewhat” or “a little” knowledgeable of the concepts of sex/gender prior to the workshop. Overall, participants rated the readability and content of the briefing notes highly (median 6.0 [range 2–7]; median 6.0 [range 1–7] respectively). Participants indicated that the issue of sex/gender analysis was highlighted clearly (median 6 [range 4–7]) and that the concepts of sex and gender and sex/gender analysis were well defined (median 6 [range 2–7]). Defining the concepts of sex/gender was generally considered the most challenging aspect of communicating about sex/gender analysis, and participants suggested the addition of examples to illustrate the non-discrete and complex characteristics of gender identity and other elements relevant to gender, such as marginalization and other dynamics of power relations:

“There needs to be more emphasis on the continuum of gender to promote inclusion of marginalized groups (e.g., transgendered, asexual)”.

Participants indicated that the briefing notes provided clear methodological guidance to address sex/gender in reviews (median 6 [range 3–7]). However, the written feedback on the methods section was mixed with some participants noting that this section was “too complex” and others stating that it was “the most helpful”. Despite these limitations, participants reported that the examples provided were helpful in illustrating how to apply the concepts (median 6 [range 3–7]):

“The illustrations certainly help to contribute to a more complete and deeper understanding of [the] sex and gender issue”.

Some participants suggested that additional examples were warranted or existing ones could be clarified by describing a wider breadth of the characteristics and experiences that may be associated with gender:

“When criteria are mentioned such as age and ethnicity, [one] could also include socioeconomic status and for this health issue [HIV], involvement in sex trade, experiences of sexual violence”.

Importantly, the majority of participants (89%) strongly agreed that the application of this guidance related to sex/gender would add value to a Cochrane systematic review (median 7 [range 4–7]). However, 79% of participants thought that there were potential barriers to incorporating sex/gender into systematic reviews. Participants cited the lack of access to data to conduct such analyses and the additional work required particularly in areas where evidence of the importance of sex/gender was limited:

“Most RCTs don’t report sex disaggregated data”.

“You can try to apply all questions of sex and gender to each review but authors are limited to the data recorded. Even if they approach clinical trial authors, much of this information will not be available”.

“Adds complexity that may not be welcome particularly in areas with less overt sex/gender issues”.

Others cited conceptual challenges, the need for additional skills, and barriers related to the varied beliefs among reviewers about the importance of sex/gender:

“Gender is very subjective and difficult to capture”.

“I think the biggest barrier will be the complexity around applying a gender lens to systematic reviews. It requires us to think ‘out of our boxes’ at times. Gender analysis requires cultural context which adds a layer of complexity”.

“Those doing systematic reviews may not ‘want to’ apply this framework. I wouldn’t want to see the review not get done at all because a researcher doesn’t want to incorporate the sex/gender analysis or because the data is too scarce to do gender analysis”.

“Some reviewers may not see the need (different beliefs); may be controversial, careful wording of gender will be important”.

Despite noting these challenges, participants agreed that sex/gender analysis is a necessary addition to systematic reviews and that the briefing notes provided a useful approach to addressing sex/gender in reviews. They noted that the inclusion of examples targeted at specific content areas (e.g. HIV/AIDS) assists end-users in understanding the merits of sex/gender analysis and may facilitate the uptake of sex/gender analysis in systematic reviews.

## Discussion

This pilot study demonstrated that systematic review producers and users welcomed guidance on sex/gender analysis in the form of briefing notes. Participants identified the need for further examples of sex/gender analysis in systematic reviews with consideration of other intersecting characteristics (such as cultural context), and the need to assess and overcome barriers such as lack of sex/gender analysis in primary research and increased workload of applying a gender lens to systematic reviews.

Most reviewers seem to want to learn sex/gender analysis and apply this information to reviews despite the barriers identified. However, the barriers deserve further discussion. First, there is considerable complexity in the concept of ‘sex/gender’ which is purposely used to reflect the interrelationships between the biological processes of sex (e.g., the biological, genetic and physiological processes that generally distinguish males and females), and the social processes of gender (e.g., gender roles, relationships, relative power and other traits that societies generally ascribe to men and women and people of diverse gender identities), [4] and which cannot be neatly measured or disaggregated. Researchers have begun to address this complexity through exploring the inclusion of qualitative data in reviews and the use of narrative summaries [55]. These approaches may assist in providing methodological guidance on how best to incorporate concepts, such as sex/gender, that do not readily lend themselves to quantification. In fact, the need for methods and tools to contextualise evidence for diverse populations and settings are increasingly being addressed in systematic review methodology.

Second, the extent of sex/gender analysis applied to each systematic review will depend on the review question and what is appropriate and/or feasible for that question. For example, researchers considering the effectiveness of total joint arthroplasty (TJA) may decide to report outcomes by sex to determine whether TJA has different benefit-harm ratios for men and women. These reviewers may also justify this methodological decision in the background of their review by highlighting the rich literature on sex/gender and TJA treatment decisions [56], wait times [57] or symptoms [58]. Or reviewers may decide to contextualize or discuss their findings in the implications section of their review by highlighting the potential practice implications of their findings for men and women [59]. Additionally, our guidance in the briefing notes encourages systematic review authors to report both what is known and not known about the sex/gender implications of their review question. In this way, gaps in knowledge are highlighted, with potential to influence future research agendas, and issues such as a lack of data availability to answer sex/gender-related research questions are documented rather than omitted. The lack of access to data to conduct sex-disaggregated analysis is a challenge cited not only by participants in this pilot project but also by other systematic reviewers [25], [60], [61]. The briefing notes advise authors to contact primary study authors for additional data and to transparently report on the outcome of these requests. As cited by some of the workshop participants who assessed the briefing notes, these requests for data are not always fruitful and can add additional work. Finally, while, workshop participants agreed that sex/gender analysis would add value to a systematic review, many were concerned about the risk of drawing spurious inferences from subgroup analyses, underscoring the need to use appropriate methodology, in alignment with concerns expressed by other investigators [31], [62].

While sex/gender analysis may not be appropriate for all review questions, there may be potential consequences of not conducting such analyses. For example, between 1997 and 2001, eight of the ten prescription drugs withdrawn from market by the U.S. Food and Drug Administration (FDA) were associated with more harm among women as compared to men, either because women were more susceptible to adverse effects or because more women were prescribed the drug [63]. Furthermore, in a 2014 landmark decision, the FDA, for the first time, recommended different dosages of a drug for men and women due to increased adverse effects for women [64]. Availability of sex-disaggregated data may have alerted prescribers and consumers earlier to there being a greater risk for women or gaps in safety evidence for women.

This pilot project has limitations that we aim to address in subsequent research. First, our preliminary evaluation was based on a relatively small number of persons, self-selected to attend a Canadian-based meeting. A more comprehensive evaluation of the briefing notes is needed to assess their impact on the appropriate consideration of sex/gender in systematic reviews, which we plan to conduct once their full dissemination is underway. It will be important to determine how and in what ways the briefing notes have made an impact on the uptake of sex/gender analysis in systematic reviews. Second, because we are affiliated with the Cochrane Collaboration we worked solely with Cochrane review groups, editors and conference attendees on the design and evaluation of the briefing notes. While the Cochrane Collaboration is a lead producer of systematic reviews internationally, further evaluation and dissemination of the briefing notes throughout the Cochrane Collaboration network and beyond Cochrane is needed to ensure wider representation of systematic review authors. Through wider dissemination and evaluation efforts we hope to engage more members of the systematic review community in the development of methodological and practical guidance to facilitate sex/gender analysis and foster changes in practice.

## Conclusion

Sex/gender analysis is important to identify the differential effects of interventions for men and women and address to whom the evidence does, does not, or may not apply. Integrating sex/gender analysis in systematic reviews can enhance the quality of health evidence, with the goals of improved health care quality and health outcomes for all. Our team, working collaboratively with three Cochrane Systematic Review Groups, has produced a pilot series of targeted and tailored briefing notes to support the consideration of sex/gender in systematic reviews and guide reviewers on how to undertake sex/gender analysis in evidence synthesis. Our evaluation demonstrates that briefing notes have the potential to be an effective knowledge translation tool to increase awareness and consideration of sex/gender in systematic reviews.

## Supporting Information

Appendix S1 Briefing Note: Cochrane Musculoskeletal Group: Addressing Sex/Gender in Systematic Reviews.
(DOC)
