# Supplementary material for: Development and Evaluation of ‘Briefing Notes’ as a Novel Knowledge Translation Tool to Aid the Implementation of Sex/Gender Analysis in Systematic Reviews: A Pilot Study
Source: PLoS One. 2014 Nov 5;9(11):e110786. doi: 10.1371/journal.pone.0110786 (PMC4220945; doi:10.1371/journal.pone.0110786)
Supplement: Appendix S1 — Briefing Note: Cochrane Musculoskeletal Group: Addressing Sex/Gender in Systematic Reviews. (DOC) [file pone.0110786.s001.doc]

*The purpose of this topic-specific briefing note is to provide systematic review authors with information and guidance on sex and gender analysis.*

1. **Why address sex and gender in systematic reviews?**

- The safety and effectiveness of health interventions can differ within and between populations of men and women for many reasons including issues of sex and gender.
- There is consensus amongst various stakeholders (e.g., journal editors, research funders, policy-makers) that the consideration of sex and gender in research is not only essential for scientific rigour but also for informed decision-making, for reduction of harm and for addressing inequities in health.
- There is, however, a lack of consistent analysis and reporting about sex and gender in health research (including primary studies and systematic reviews), and continued underrepresentation of women in some clinical trials ([Pinnow et al., 2014](http://www.ncbi.nlm.nih.gov/pubmed/24405314); [Health Canada, 2013](http://www.hc-sc.gc.ca/dhp-mps/prodpharma/applic-demande/guide-ld/clini/womct_femec-eng.php); [Doull et al., 2010](http://www.ncbi.nlm.nih.gov/pubmed/20384450); [Jagsi et al., 2009](http://www.ncbi.nlm.nih.gov/pubmed/19507175); [Pinnow et al., 2009](http://www.fda.gov/downloads/ScienceResearch/SpecialTopics/WomensHealthResearch/UCM247209.pdf)).
- Review authors and editors have identified a need for guidance on the integration of sex/gender analysis (SGA) into systematic review methods.
- As a leader in systematic review methodology, the Cochrane Collaboration must continue to advance the evidence-base and its applicability by integrating sex/ gender analysis into its reviews.

**Applying sex/gender analysis: ‘A few pointers’**

- **The terms ‘sex’ and ‘gender’ are not interchangeable,** but rather, the pathways between these processes should be explored and documented.

- At an individual level, one’s **sex** is “embedded” within one’s **gender**. Explanations of **sex** differences must therefore consider the intersection of sex/gender.

**-** Even when health issues are sex-specific (e.g., prostate cancer, ovarian cancer), dynamics of **gender** may affect prevention, how and when care is sought, diagnosis, treatment and outcome.

-  **Sex-disaggregation** (reporting results for males and females separately) **is a necessary but not sufficient step** for sex/gender analysis.

- Sex/gender can interact with **other determinants of health** ([PHAC, 2013](http://www.phac-aspc.gc.ca/ph-sp/determinants/determinants-eng.php#secondreport)) to influence health status and the effectiveness of interventions.

- Gender/sex is part of the acronym **PROGRESS-**Plus ([Evans & Brown, 2003](http://www.ncbi.nlm.nih.gov/pubmed/12772480); [Welch et al., 2012](http://www.plosone.org/article/info%3Adoi%2F10.1371%2Fjournal.pone.0031360)) used by the Campbell and Cochrane Equity Group to list socio-demographic factors across which differences in health or therapies may be considered inequitable.

1. **What are ‘sex’, ‘gender’, and ‘SGA’?**

- **Sex** refers to the biological, genetic and physiological processes that generally distinguish females from males.
- **Gender** refers to the roles, relationships, behaviours, relative power, and other traits that societies generally ascribe to women, men, and people of diverse gender identities (e.g., transgender persons).
- Although often categorized as binary for analysis (e.g., male/female; masculine/feminine), attributes of sex and gender are multidimensional, dynamic and interactive. The term **sex/gender** is used to highlight this ‘entanglement’ of the biological and the social ([Kaiser et al, 2009](http://www.ncbi.nlm.nih.gov/pubmed/19406148); [Springer et al, 2011](http://www.ncbi.nlm.nih.gov/pubmed/21724313); [Tudiver et al, 2012](http://www.cihr-irsc.gc.ca/e/44734.html#a04)).
- **Sex/gender analysis** is an analytic framework that is used to explore possible biological and social similarities and differences between and among men and women. In the context of health care systems, SGA explores the interrelationships of sex and gender within or between groups in order to identify how these may affect health experiences, access to care, and health outcomes.

1. **Why does addressing sex and gender in systematic reviews of musculoskeletal interventions matter?**

- Some musculoskeletal (MSK) disorders have different prevalence in men and women. Often this reflects underlying pathophysiologic mechanisms. For example, both innate and adaptive immunity differ between men and women, contributing to differences in the prevalence of autoimmune states such as rheumatoid arthritis, systemic lupus erythematosis (SLE) and Sjogren's syndrome (more common in women). Other conditions have higher prevalence in men (e.g., gout; ankylosing spondylitis).
- Choice of diagnostic criteria may also favor diagnosis in one sex (e.g., the original American College of Rheumatology (ACR) criteria for fibromyalgia favor diagnosis in women more than the updated 2010 criteria do).
- Sex/gender may affect the severity and progression of musculoskeletal conditions or their complications (e.g., SLE; spondyloarthritis; rheumatoid arthritis; knee osteoarthritis).
- Overall functional capacity and quality of life, and the ways a disease or MSK injury are experienced (e.g., the perception of pain) can be influenced by sex/gender.
- Differences in immunity, other pathophysiologic mechanisms, or pharmacokinetics and pharmacodynamics may result in differential responses of men and women to interventions.
- Sex/gender can also affect adherence, the ability to access health care, health care provider responses to individuals (including diagnosis and treatments offered) and social support networks.
- In some situations, it is not clear whether sex/gender differences are multifactorial or predominantly due to inherent differences in the biology of a disease, confounders (e.g., size of bones), the manner in which men and women respond to treatment, or the therapies given.
- The presentation of sex-disaggregated data often illuminates differences and similarities as a starting point but a contextual analysis that considers the intersections between sex, gender and other health determinants is needed.

**4.** **What can – and should – be done about it?**

- Reviewers should **consider and document whether and in what ways sex and/or gender are relevant** to their review question.
- **Depending on the review question, different (or multiple) methods may be appropriate.** The following table provides some strategies to address issues of sex/gender in reviews and examples to illustrate some of the ways in which sex and/or gender can play a role in MSK disorders.
- **In the table, sex/gender are dichotomized intentionally for clarity but emerging theory highlights the intersections between the two.**

| **Methods** | **Guidance** |
| --- | --- |
| Question formulation | - **Consider whether there are known or possible differences by sex/gender across: baseline risk, prevalence, vulnerability, implementation or response to intervention, and plan objectives and methods accordingly.** - **Example:** Women have a higher incidence and prevalence of knee osteoarthritis, which is more severe than in men ([Srikanth et al., 2005](http://www.ncbi.nlm.nih.gov/pubmed/15978850)). - **Example:** In a systematic review on quality of life after total hip and total knee arthroplasty, men appeared to benefit more from the intervention in the few studies that addressed this issue ([Ethgen et al., 2004](http://www.ncbi.nlm.nih.gov/pubmed/15118039)). |
| Context | - **Consider sociocultural understandings of sex/gender as part of context** [e.g., how gender roles, norms, identity, and gender relations can influence symptoms, treatment implementation and health]. - This may include developing an **analytic framework/logic model** to define assumptions about sex/gender across intervention, comparator and outcomes. |
| **P**opulation | - **Specify if sex/gender will be used as a basis for exclusion or inclusion of studies.** - **Consider ways in which other inclusion criteria at the review or primary study level, such as age or ethnicity, may interact with sex/gender.** For example, age of onset of disease or complications related to a condition may differ for men and women – thus age-based inclusion criteria may favour one sex. |
| **I**ntervention  **C**omparator | - **Consider whether sex/gender interacts with aspects of the intervention (e.g. treatment delivery; likelihood of offering treatment; acceptability),** and whether this is important for the review question. - **Example:** Men and women are known to experience different quality of health care for osteoarthritis because men are more likely to be offered total knee replacement surgery. In a review assessing disparities in osteoarthritis treatment across sex and other factors, only one study assessed differences in health care quality across sex ([Borkhoff et al., 2008](http://www.cmaj.ca/content/178/6/681.full)). |
| **O**utcomes | - **Consider whether the outcomes are relevant for both women and men. Does the condition or its complications manifest differently in men and women – and do the selected outcomes for the review capture both of these scenarios?** - **Example:** Peripheral arthritis may be more common in women with inflammatory arthritides whereas men may experience more inflammatory back pain so consider capturing both outcomes ([Barnabe et al., 2012](http://www.ncbi.nlm.nih.gov/pubmed/22505697)). |
| Study design | - **Consider study design eligibility according to the review question and objectives related to sex and gender. If** [**non-randomized studies**](http://handbook.cochrane.org/chapter_13/13_including_non_randomized_studies.htm) **are considered, why, and what are their eligible features?** - **Example:** A systematic review that included cohort studies as well as RCTs on men and women with inflammatory arthritis reported sex differences in pain. Differences may be multifactorial (e.g., delay in referral, more severe or aggressive disease, psychosocial factors). The authors concluded that it is important to consider implications of sex differences in classifying responses and that future studies should recognize the need to evaluate for differential baseline pain levels and treatment responses ([Sun et al., 2012](http://www.bmj.com/content/344/bmj.e1553)). |
| Searching for studies | - Indexing for studies that assess sex/gender is not consistent. Applying these terms to a search may unnecessarily limit the scope, unless the search terms have been validated. Searching beyond usual databases may be required to assess influence of gender and sociocultural context. |
| Data collection | - **Extract sex-disaggregated data;** contact authors for sex of participants and sex-disaggregated data if not reported. - **Identify and report characteristics of the population that might intersect with sex/gender** (e.g. PROGRESS Plus factors). |
| Risk of bias | - Be aware that a potential form of **reporting bias** would occur if only a subset of studies provided sex-disaggregated data so that findings were based only on the subset. |
| Data analysis | - **Subgroup Analysis:** Specify the method of subgroup analysis by sex/gender in the protocol, with attention to credibility criteria for subgroup analyses ([Sun et al., 2012](http://www.ncbi.nlm.nih.gov/pubmed/22422832); also [see Cochrane Handbook](http://handbook.cochrane.org/chapter_9/9_6_2_what_are_subgroup_analyses.htm)). - **Sensitivity Analysis:** Conduct pre-planned sensitivity analysis to assess the robustness of results across sex/gender. - **Heterogeneity:** Consider sex/gender as reasons for heterogeneity and explore with methods such as meta-regression techniques and subgroup analyses ([see Cochrane Handbook](http://handbook.cochrane.org/chapter_9/9_5_heterogeneity.htm)). - Consider the use of [individual patient data meta-analysis](http://handbook.cochrane.org/chapter_18/18_reviews_of_individual_patient_data.htm) to assess differences in effects across sex/gender. - Be aware of [ecological bias](http://handbook.cochrane.org/chapter_9/9_6_5_5_be_aware_that_the_effect_of_a_characteristic_may_not.htm) (aggregation bias, ecological fallacy) with meta-regression techniques. - Consider effects of possible confounders of sex/gender differences (e.g., age, ethnicity, bone size and others). |
| Additional analyses | - Consider additional analyses and **qualitative methods** such as realist review ([Thomas et al., 2008](http://www.biomedcentral.com/1471-2288/8/45)), meta-ethnography ([Pawson et al., 2005](http://www.ncbi.nlm.nih.gov/pubmed/16053581)), and thematic analysis ([Atkins et al., 2008](http://www.biomedcentral.com/1471-2288/8/21)) to address or situate results within sociological understandings of gender, if relevant to the question of interest. |
| Presenting results and summary of findings | - **Report number and percentage of both female and male participants** in included studies and report if data are not available. - Report **results of planned analyses related to sex/gender**, and justify why any planned analyses were not conducted. - Identify and **justify any post hoc, exploratory analyses** related to sex/gender. - Consider whether a separate **summary of findings table** is needed to show differences/similarities in response or baseline event rate across sex/gender. |
| Interpreting results and drawing conclusions | - **Describe to whom the available evidence applies,** and the implications for the overall quality of the findings in relation to sex/gender and any intersecting characteristics. - Outline **research gaps or unanswered questions** related to sex and gender analysis, and identify planned analyses that could not be conducted due to unavailable data. - **Example:** In a review on cognitive behavioural therapies for fibromyalgia, efficacy was demonstrated in adults. However, the majority of patients were middle-aged Caucasian women, making it difficult to apply the results to all patients, especially to male or non-Caucasian patients. No study performed subgroup analyses by sex or race/ethnicity ([Bernardy et al, 2013](http://www.ncbi.nlm.nih.gov/pubmed/24018611)). |

**Questions? Comments?**

- The Sex/Gender Methods Group, affiliated with the Campbell and Cochrane Equity Methods Group, is available to support authors in implementing this guidance.
- This document may be used and distributed. We would appreciate the following citation: Welch, V., Puil L., Shea B., Runnels V., Doull M., Tudiver S., Boscoe M for the Sex/Gender Methods Group. Addressing Sex/Gender in Systematic Reviews: Cochrane Musculoskeletal Group Briefing Note. Version 2014-01. Accessed at: http://equity.cochrane.org/sex-and-gender-analysis.
- Web link for resources: <http://equity.cochrane.org/sex-and-gender-analysis>
- Contact: [Jennifer.Petkovic@uottawa.ca](mailto:Jennifer.Petkovic@uottawa.ca)

**References**

Atkins S. Lewin S, Smith H, Engel M, Fretheim A, Volmink J. Conducting a meta-ethnography of qualitative literature: Lessons learnt. BMC Medical Research Methodology 2008; 8:21 doi:10.1186/1471-2288-8-21

[Barnabe C](http://www.ncbi.nlm.nih.gov/pubmed?term=Barnabe C%5BAuthor%5D&cauthor=true&cauthor_uid=22505697), [Bessette L](http://www.ncbi.nlm.nih.gov/pubmed?term=Bessette L%5BAuthor%5D&cauthor=true&cauthor_uid=22505697), [Flanagan C](http://www.ncbi.nlm.nih.gov/pubmed?term=Flanagan C%5BAuthor%5D&cauthor=true&cauthor_uid=22505697), [Leclercq S](http://www.ncbi.nlm.nih.gov/pubmed?term=Leclercq S%5BAuthor%5D&cauthor=true&cauthor_uid=22505697), [Steiman A](http://www.ncbi.nlm.nih.gov/pubmed?term=Steiman A%5BAuthor%5D&cauthor=true&cauthor_uid=22505697), [Kalache F](http://www.ncbi.nlm.nih.gov/pubmed?term=Kalache F%5BAuthor%5D&cauthor=true&cauthor_uid=22505697), [Kung T](http://www.ncbi.nlm.nih.gov/pubmed?term=Kung T%5BAuthor%5D&cauthor=true&cauthor_uid=22505697), [Pope JE](http://www.ncbi.nlm.nih.gov/pubmed?term=Pope JE%5BAuthor%5D&cauthor=true&cauthor_uid=22505697), [Haraoui B](http://www.ncbi.nlm.nih.gov/pubmed?term=Haraoui B%5BAuthor%5D&cauthor=true&cauthor_uid=22505697), [Hochman J](http://www.ncbi.nlm.nih.gov/pubmed?term=Hochman J%5BAuthor%5D&cauthor=true&cauthor_uid=22505697), [Mosher D](http://www.ncbi.nlm.nih.gov/pubmed?term=Mosher D%5BAuthor%5D&cauthor=true&cauthor_uid=22505697), [Thorne C](http://www.ncbi.nlm.nih.gov/pubmed?term=Thorne C%5BAuthor%5D&cauthor=true&cauthor_uid=22505697), [Bykerk V](http://www.ncbi.nlm.nih.gov/pubmed?term=Bykerk V%5BAuthor%5D&cauthor=true&cauthor_uid=22505697). Sex differences in pain scores and localization in inflammatory arthritis: a systematic review and metaanalysis J Rheumatol. 2012 Jun;39(6):1221-30. doi: 10.3899/jrheum.111393. Epub 2012 Apr 15.

Bernardy K, Klose P, Busch AJ, Choy EHS, Häuser W. Cognitive behavioural therapies for fibromyalgia. Cochrane Database of Systematic Reviews 2013; Issue 9. Art. No.: CD009796. DOI: 10.1002/14651858.CD009796.pub2.

Borkhoff CM Hawker G, Kreder HJ, Glazier RH, Mahomed NN, Wright JG. The effect of patients' sex on physicians' recommendations for total knee arthroplasty. CMAJ March 11, 2008; 178 (6). doi:10.1503/cmaj.071168

Doull, M, Runnels V, Tudiver S, Boscoe M. Appraising the evidence: applying sex and gender based analysis (SGBA) to Cochrane systematic reviews on cardiovascular diseases. Journal of Women's Health 2010. 19(5):997-1003.

[Ethgen O](http://www.ncbi.nlm.nih.gov/pubmed?term=Ethgen O%5BAuthor%5D&cauthor=true&cauthor_uid=15118039), [Bruyère O](http://www.ncbi.nlm.nih.gov/pubmed?term=Bruyère O%5BAuthor%5D&cauthor=true&cauthor_uid=15118039), [Richy F](http://www.ncbi.nlm.nih.gov/pubmed?term=Richy F%5BAuthor%5D&cauthor=true&cauthor_uid=15118039), [Dardennes C](http://www.ncbi.nlm.nih.gov/pubmed?term=Dardennes C%5BAuthor%5D&cauthor=true&cauthor_uid=15118039), [Reginster JY](http://www.ncbi.nlm.nih.gov/pubmed?term=Reginster JY%5BAuthor%5D&cauthor=true&cauthor_uid=15118039). Health-related quality of life in total hip and total knee arthroplasty. A qualitative and systematic review of the literature. J Bone Joint Surg Am 2004 May;86-A(5):963-74.

Evans T, Brown H. Road traffic crashes: operationalizing equity in the context of health sector reform. Inj Control Saf Promot 2003; 10: 11-2.

Health Canada (2013).  Guidance Document: Considerations for Inclusion of Women in Clinical Trials and Analysis of Sex Differences. Published by authority of the Minister of Health. 2013/5/29. Available at: <http://www.hc-sc.gc.ca/dhp-mps/prodpharma/applic-demande/guide-ld/clini/womct_femec-eng.php>. Last accessed Jan 24, 2014.

Higgins JPT, Green S (editors). Cochrane Handbook for Systematic Reviews of Interventions Version 5.0.2 [updated September 2009]. The Cochrane Collaboration, 2009. Available from [www.cochrane-handbook.org](http://www.cochrane-handbook.org/).

Jagsi R, Motomura AR, Amarnath S, Jankovic A, Sheets N, Ubel PA. Under-representation of women in high-impact published clinical cancer research. Cancer. 2009 Jul 15;115(14):3293-301. doi: 10.1002/cncr.24366.

Kaiser, A, Haller, S, Schmitz, S, Nitsch, C. On sex/gender related similarities and differences in fMRI language research. Brain Research Review 2009; 61(2): 49e59.

Pawson R, Greenhalgh T, Harvey G, Walshe K. Realist review – a new method of systematic review designed for complex policy interventions. Journal of Health Services & Policy 2005; Vol 10 Suppl1: 21-34.

Pinnow E, Sharma P, Parekh A, Gevorkian N, Uhl K. Increasing participation of women in early phase clinical trials approved by the FDA. Women’s Health Issues 2009; 19:89–93.

Pinnow E, Herz N, Loyo-Berrios N, Tarver M. Enrollment and Monitoring of Women in Post-Approval Studies for Medical Devices Mandated by the Food and Drug Administration. J Womens Health (Larchmt). 2014 Jan 9. [Epub ahead of print]

Public Health Agency of Canada. What Makes Canadians Healthy or Unhealthy? Revised January 15, 2013. [http://www.phac-aspc.gc.ca/ph-sp/determinants/determinants-eng.php#secondreport](http://www.phac-aspc.gc.ca/ph-sp/determinants/determinants-eng.php#secondreport). Accessed on April 3, 2013.

Springer KW, Stellman JM, Jordan-Young RM. Beyond a catalogue of differences: a theoretical frame and good practice guidelines for researching sex/gender in human health. Social Science & Medicine 2011; 1-8. doi.10.1016/j.socscimed.2011.05.033.

[Srikanth VK](http://www.ncbi.nlm.nih.gov/pubmed?term=Srikanth VK%5BAuthor%5D&cauthor=true&cauthor_uid=15978850), [Fryer JL](http://www.ncbi.nlm.nih.gov/pubmed?term=Fryer JL%5BAuthor%5D&cauthor=true&cauthor_uid=15978850), [Zhai G](http://www.ncbi.nlm.nih.gov/pubmed?term=Zhai G%5BAuthor%5D&cauthor=true&cauthor_uid=15978850), [Winzenberg TM](http://www.ncbi.nlm.nih.gov/pubmed?term=Winzenberg TM%5BAuthor%5D&cauthor=true&cauthor_uid=15978850), [Hosmer D](http://www.ncbi.nlm.nih.gov/pubmed?term=Hosmer D%5BAuthor%5D&cauthor=true&cauthor_uid=15978850), [Jones G](http://www.ncbi.nlm.nih.gov/pubmed?term=Jones G%5BAuthor%5D&cauthor=true&cauthor_uid=15978850). A meta-analysis of sex differences prevalence, incidence and severity of osteoarthritis. Osteoarthritis Cartilage 2005 Sep;13(9):769-81.

[Sun X](http://www.ncbi.nlm.nih.gov/pubmed?term=Sun X%5BAuthor%5D&cauthor=true&cauthor_uid=22422832), [Briel M](http://www.ncbi.nlm.nih.gov/pubmed?term=Briel M%5BAuthor%5D&cauthor=true&cauthor_uid=22422832), [Busse JW](http://www.ncbi.nlm.nih.gov/pubmed?term=Busse JW%5BAuthor%5D&cauthor=true&cauthor_uid=22422832), [You JJ](http://www.ncbi.nlm.nih.gov/pubmed?term=You JJ%5BAuthor%5D&cauthor=true&cauthor_uid=22422832), [Akl EA](http://www.ncbi.nlm.nih.gov/pubmed?term=Akl EA%5BAuthor%5D&cauthor=true&cauthor_uid=22422832), [Mejza F](http://www.ncbi.nlm.nih.gov/pubmed?term=Mejza F%5BAuthor%5D&cauthor=true&cauthor_uid=22422832), [Bala MM](http://www.ncbi.nlm.nih.gov/pubmed?term=Bala MM%5BAuthor%5D&cauthor=true&cauthor_uid=22422832), [Bassler D](http://www.ncbi.nlm.nih.gov/pubmed?term=Bassler D%5BAuthor%5D&cauthor=true&cauthor_uid=22422832), [Mertz D](http://www.ncbi.nlm.nih.gov/pubmed?term=Mertz D%5BAuthor%5D&cauthor=true&cauthor_uid=22422832), [Diaz-Granados N](http://www.ncbi.nlm.nih.gov/pubmed?term=Diaz-Granados N%5BAuthor%5D&cauthor=true&cauthor_uid=22422832), [Vandvik PO](http://www.ncbi.nlm.nih.gov/pubmed?term=Vandvik PO%5BAuthor%5D&cauthor=true&cauthor_uid=22422832), [Malaga G](http://www.ncbi.nlm.nih.gov/pubmed?term=Malaga G%5BAuthor%5D&cauthor=true&cauthor_uid=22422832), [Srinathan SK](http://www.ncbi.nlm.nih.gov/pubmed?term=Srinathan SK%5BAuthor%5D&cauthor=true&cauthor_uid=22422832), [Dahm P](http://www.ncbi.nlm.nih.gov/pubmed?term=Dahm P%5BAuthor%5D&cauthor=true&cauthor_uid=22422832), [Johnston BC](http://www.ncbi.nlm.nih.gov/pubmed?term=Johnston BC%5BAuthor%5D&cauthor=true&cauthor_uid=22422832), [Alonso-Coello P](http://www.ncbi.nlm.nih.gov/pubmed?term=Alonso-Coello P%5BAuthor%5D&cauthor=true&cauthor_uid=22422832), [Hassouneh B](http://www.ncbi.nlm.nih.gov/pubmed?term=Hassouneh B%5BAuthor%5D&cauthor=true&cauthor_uid=22422832), [Walter SD](http://www.ncbi.nlm.nih.gov/pubmed?term=Walter SD%5BAuthor%5D&cauthor=true&cauthor_uid=22422832), [Heels-Ansdell D](http://www.ncbi.nlm.nih.gov/pubmed?term=Heels-Ansdell D%5BAuthor%5D&cauthor=true&cauthor_uid=22422832), [Bhatnagar N](http://www.ncbi.nlm.nih.gov/pubmed?term=Bhatnagar N%5BAuthor%5D&cauthor=true&cauthor_uid=22422832), [Altman DG](http://www.ncbi.nlm.nih.gov/pubmed?term=Altman DG%5BAuthor%5D&cauthor=true&cauthor_uid=22422832), [Guyatt GH](http://www.ncbi.nlm.nih.gov/pubmed?term=Guyatt GH%5BAuthor%5D&cauthor=true&cauthor_uid=22422832). Credibility of claims of subgroup effects in randomised controlled trials: systematic review. BMJ 2012 Mar 15;344:e1553. doi: 10.1136/bmj.e1553.

Thomas J, Harden A. ESRC National Centre for Research Methods NCRM Working Paper Series Number (10/07).Methods for the thematic synthesis of qualitative research in systematic reviews. http://eppi.ioe.ac.uk/cms/Default.aspx?tabid=188

Tudiver, S, Boscoe, M, Runnels, VE, Doull, M[. Challenging "dis-ease": sex, gender and systematic reviews in health](http://www.cihr-irsc.gc.ca/e/44734.html#a04) in What a difference sex and gender make: A Gender, Sex and Health Research Casebook. Ottawa: Canadian Institutes of Health Research, Institute of Gender and Health 2012.

Welch V, Petticrew M, Ueffing E, Benkhalti Jandu M, Brand K, et al. Does Consideration and Assessment of Effects on Health Equity Affect the Conclusions of Systematic Reviews? A Methodology Study. PLoS ONE 2012; 7(3): e31360. doi:10.1371/journal.pone.0031360
